# DNA methylation at individual CpG-sites of *EPB41L3*, *HTERT* and *FAM19A4* are useful for detection of cervical high-grade squamous intraepithelial lesions (HSIL) or worse: Analysis of individual CpG-sites outperforms averaging

**DOI:** 10.1016/j.tvr.2024.200288

**Published:** 2024-07-01

**Authors:** Monica Molano, Dorothy A. Machalek, Samuel Phillips, Grace Tan, Suzanne M. Garland, David Hawkes, Prisha Balgovind, Reza Haqshenas, Steve G. Badman, John Bolnga, Josephine Gabuzzi, Zure Kombati, Gloria M. Munnull, Julia ML. Brotherton, Marion Saville, John M. Kaldor, Pamela J. Toliman, Andrew J. Vallely, Gerald L. Murray

**Affiliations:** a Centre for Women's Infectious Diseases, The Royal Women's Hospital, Melbourne, Victoria, Australia; b Murdoch Children's Research Institute, Melbourne, Victoria, Australia; c The Kirby Institute, University of New South Wales, Sydney, NSW, Australia; d Department of Obstetrics and Gynaecology, University of Melbourne, Parkville, Victoria, Australia; e Australian Centre for the Prevention of Cervical Cancer, Melbourne, Victoria, Australia; f Papua New Guinea Institute of Medical Research, Goroka, Papua New Guinea; g Department of Obstetrics and Gynaecology, Modilon General Hospital, Madang, Papua New Guinea; h Department of Pathology, Mt Hagen Provincial Hospital, WHP 281, Papua New Guinea; i Department of Obstetrics and Gynaecology, Mt Hagen Provincial Hospital, Mount Hagen, Papua New Guinea

**Keywords:** DNA methylation, Cervical cancer, Human papillomavirus, Diagnostic test, Epigenetics, Molecular diagnostics

## Abstract

Global methylation analysis of gene promoters is promising for detection of high-grade squamous intraepithelial lesions or worse (HSIL+) in high-risk human papillomavirus (hrHPV)-positive women. However, diagnostic performance of methylation data at individual CpG-sites is limited. We explored methylation for predicting HSIL+ in self- and clinician-collected samples from Papua New Guinea.

Methylation of *EPB41L3* (1–6 CpG-sites), *hTERT* (1–10 CpG-sites) and *FAM19A4* (1–5 CpG-sites) was assessed through pyrosequencing from 44 HPV+ samples (4 cancers, 19 HSIL, 4 low-grade squamous intraepithelial lesions (LSIL), 17 normal). New primers were designed for *FAM19A4* directed to the first exon region not explored previously.

In clinician-collected samples, methylation at CpG-sites 4 and 5 of *EPB41L3* were the best HSIL predictors (AUC >0.83) and CpG-site 4 for cancer (0.925). Combination of *EPB41L3* sites 2/4 plus *FAM19A4* site 1 were the best HSIL+ markers [100% sensitivity, 63.2% specificity].

Methylation at CpG-site 5 of *FAM19A4* was the best HSIL predictor (0.67) in self-collected samples, and CpG-sites 1 and 3 of *FAM19A4* for cancer (0.77). Combined, *FAM19A4* site 1 plus HPV 16/18 detection yielded sensitivity of 82.6% and specificity of 61.9%.

In conclusion, methylation at individual CpG-sites of *EPB41L3* and *FAM19A4* outperformed global analysis and improved HSIL+ detection, warranting further investigation.

## Highlights

• Diagnostic biomarkers to triage hrHPV+ self-collected samples for detection of HSIL+ are needed.
• Methylation at CpG-sites of *EPB41L3, hTERT* and *FAM19A4* has different performance in clinician and self-collected samples.
• Methylation at individual CpG-sites improves HSIL+ detection while global methylation dampens the gene diagnostic utility.
• Methylation at individual CpG-sites could reduce overtreatment in settings using a same-day screen-and-treat program.

## List of abbreviations

**AUC**: Area under the curve
**CpG**: Cytosines that precede a guanine nucleotide
**hrHPV**: High-risk human papillomavirus
**HSIL**: High-grade squamous intraepithelial lesions
**HSIL+**: High-grade squamous intraepithelial lesion or worse
**LBC**: Liquid-based cytology
**LMIC**: Low and middle-income countries
**LSIL**: Low-grade squamous intraepithelial lesions
**PNG**: Papua New Guinea
**qPCR**: Quantitative polymerase chain reaction
**ROC**: Receiver operating characteristic
**SCC**: Squamous cell carcinoma
**VCS**: Victorian Cytology Service

## 1 Introduction

Cervical cancer is caused by a persistent infection of hrHPV. The vast majority of new cases (87%) and deaths (91%) occur in low and middle-income countries (LMICs) [1]. Recent advances in cervical screening include the switch to objective molecular testing for the detection of hrHPV types in many national screening programs [2] and the development of new strategies such as a self-sampling to attract more women into screening [3].

HPV testing is more sensitive than cytology for the detection of cervical abnormalities and of cancer. However, as most infections spontaneously resolve, not all persistently hrHPV+ women will have underlying disease. As such, referral of all positive women for treatment will result in many unnecessary procedures. In fact, first evaluations of the primary HPV screening in Europe and Australia with triage by cytology and/or HPV16/18 genotyping showed a substantial increase in colposcopy referrals compared with cytology-based screening [4,5].

While cytology is still the best strategy of triage in high income settings, it is not feasible in LMIC, which makes the stratification of hrHPV+ women who require treatment even more difficult. The World Health Organization (WHO) recommends visual assessment for immediate treatment after positive HPV DNA testing for populations living in remote areas where there are few opportunities to screen women at proper intervals and for follow-up after screening [6]. However, most HPV infections are transient, so immediate ablative treatments can lead to over treatment for women with low risk of disease [7]. Hence, the identification of novel molecular biomarkers that detect underlying disease could revolutionise screening by providing new triage tests to identify women at highest risk of cancer.

Host DNA methylation markers are a promising option for detection of HSIL lesions in hrHPV+ women in high income countries. Methylation assays have advantages over other triage strategies as they can be automated, are high throughput, have accurate quantitation, are robust to operator variations and can be performed in the same specimen as the screening hrHPV test [8]. A literature review performed by our group found that approximately 10 human genes have been evaluated in more than one study; in different clinical settings these showed consistently increased levels of methylation with increasing disease grade. Of these host methylation markers as *FAM19A4, hTERT* and *EPB41L3*, alone or in combination with other genes, have shown good performance for detection of CIN2+ among hrHPV+ women from high-income countries [[9], [10], [11], [12], [13]]. However, more basic, clinical and epidemiological information is required on the performance of these genes at individual CpG-sites in hrHPV+ women. There are substantial gaps in knowledge around marker performance in self-collected samples, and in women from LMIC where the burden of hrHPV infection and disease are high and better triage strategies are essential.

The majority of DNA methylation occurs on cytosines that precede a guanine nucleotide or CpG-sites [14,15]. Pyrosequencing is a technique that allows for relative quantitation of the base composition at each site sequenced. The ratio of C:T after bisulphite treatment indicates the proportion of unmethylated and methylated cytosines at each CpG site in the original sequence giving a more specific information of the methylation status and their possible biological and clinical role in the development of disease [14]. It is important to determine differences in methylation levels at individual CpG-sites to better define thresholds and algorithms for the detection of HSIL+ lesions. This analysis will allow us to evaluate their clinical performance individually, collectively in panels, and in combination with specific HPV typing.

The aims of the current study were: (i) To analyse DNA methylation at individual CpG-sites of *EPB41L3, hTERT and FAM19A4* genes for predicting underlying HSIL in an exploratory study of hrHPV+ women that participated in a field trial of HPV- screening-same-day-treatment in Papua New Guinea (PNG) (ii) To compare levels of methylation at individual CpG-sites in paired self/clinician-collected samples.

## 2 Methodology and analysis

### 2.1 Study population and design of the trial

We performed an exploratory study from women who participated in a field trial in PNG known as HPV-STAT, a prospective, single-arm intervention trial [16]. The trial is registered with ISRCTN, ISRCTN13476702 (https://www.isrctn.com/editorial/retrieveFile/3ed9173e-cce5-4158-a586-5777 75f0cbdd/35731). Study design, recruitment and protocols have been described [16]. Briefly, between June 5, 2018, and Jan 6, 2020, 4285 women aged 30–59 years gave informed consent and were enrolled sequentially. Inclusion and exclusion criteria were described previously [16]. A mid-cavity vaginal specimen was collected using a cytobrush (“Just for Me”, Preventative Oncology International, Cleveland Heights, Ohio) and placed into 20 ml ThinPrep PreservCyt (Hologic, Marlborough, MA). From the PreservCyt fluid 1 ml was then tested for hrHPV types using the Xpert HPV Test (GeneXpert; Cepheid, Sunnyvale, CA, USA) as per the manufacturer's instructions. HPV results were provided to women before midday to allow same-day, pelvic examination and treatment/referral. All women with a negative HPV test received their results and were advised to return to the clinic for HPV-based screening in five years.

A cervical specimen was collected by a clinician for all HPV+ women using a Cervex-Brush Combi (Rovers Medical Devices, Oss, The Netherlands), placed in 20 ml PreservCyt, and stored at 4 °C prior to shipment to Victorian Cytology Service Foundation (VCS) in Melbourne, Australia for liquid-based cytology (LBC) and p16/Ki67 dual stain cytology. A 15% random sample of HPV negative women were also asked to provide a clinician-collected cervical specimen for LBC, as above.

LBC was performed in accordance with standard operating procedures at VCS. All slides were independently assessed by two experimented cytologists and pathologists blinded to HPV-DNA test results. Where both readers agreed on a diagnosis of HSIL or worse (HSIL+), a final diagnosis was recorded. If the assessment differed, dual p16/Ki-67 immuno-staining was performed by using CINTec PLUS Cytology (Roche Diagnostics, Rotkreuz, Switzerland) to make a final diagnosis [16]. LBC was the reference standard rather than histology (gold reference in high-resource settings). It is not currently feasible to provide colposcopy examination or to collect cervical biopsies for histological analysis in PNG due limited specialist staff and infrastructure.

### 2.2 Participants in the exploratory study and design

The exploratory study included 44 hrHPV+ paired cervical and vaginal samples from women that participated in the HPV screening-same day treatment trial at Mt Hagen General Hospital (Mount Hagen, Western Highlands Province). This included all 23 hrHPV+ HSIL or SCC (19 HSIL and 4 SCC) cases identified on LBC conducted at VCS by the end of 2018 and 21 randomly selected hrHPV+ normal/LSIL samples (17 normal LBC and 4 LSIL). The choice of this sample size was based on methylation data published that showed through simulation studies and real data from the NCBI Gene Expression Omnibus, that at least 12 specimens in each group is needed to detect truly differential DNA methylation with a power ≥80%, reproducible results and consistent when using different statistical methods [15].

Molecular biologists and technicians were blinded to point of care HPV results and clinical diagnosis during the performance of the different molecular assays.

### 2.3 DNA extraction and HPV specific typing

These molecular analyses were carried out at the Royal Women's Hospital, in Melbourne, Australia. DNA was extracted by using the MagNA Pure 96 System (DNA and Viral Nucleic Acid Small Volume Kit; Roche Molecular Diagnostics; Mannheim, Germany) as per the manufacturer's Pathogen Universal 200 protocol, and eluted in 100 μL. DNA concentration was quantitated by Qubit® Fluorometer (Life technologies, California, USA). Extracted DNA was assessed for integrity by quantitative polymerase chain reaction (PCR) amplification of a 260 base-pair product of the human beta-globin gene [17].

HPV genotyping was performed using Anyplex II HPV HR14 HPV detection multiplex assay (Seegene, Seoul, South Korea), which detects 14 oncogenic HPV types (16, 18, 31, 33, 35, 39, 45, 51, 52, 56, 58, 59, 66, 68) and an internal control according to manufacturer's recommendations.

### 2.4 Bisulphite modification

DNA extracted from clinician and self-collected samples (1–100 ng) and a SiHa cervical cell line control (1–2 copies of HPV16 per cell, ATCC Cat# HTB-35, RRID:CVCL_0032; American Type Culture Collection (ATCC), Manassas, Virginia, USA; 100 ng) were bisulphite treated using Methylamp DNA modification Kit (Epigentek, Brooklyn, NY, USA) as per the manufacturer's instructions. Modified DNA was eluted in 40 μL of the Methylamp elution buffer.

### 2.5 DNA methylation analysis: PCR amplification and pyrosequencing for *EPB41L3*, *FAM19A4* and *hTERT* genes

Individual PCR targeting specific CpG-sites of *EPB41L3, FAM19A4* and *hTERT* genes were performed using the converted DNA. *EPB41L3* and *hTERT* amplifications were performed as previously described [18] with minor modifications. Briefly, 12.5 μl of Hot Start Taq Master mix (Qiagen, Valencia, CA), 0.25 pmol of each primer, 8.25 μl of water and 3 μl sample were mixed for each reaction. For *FAM19A4* amplification, 0.5 pmol of each primer and 7 μl of water were used. PCR conditions were: 95 °C for 15 min and then 45 cycles: 30 s at 94 °C; 30 s at the optimized primer-speciﬁc annealing temperature (*EPB41L3*, 56 °C, *hTERT*, 58 °C*, FAM19A4*, 50 °C), 30 s at 72 °C and a ﬁnal extension for 10 min at 72 °C. Sequences of the primers and characteristics of the amplified products are shown in Table 1. Amplification was confirmed by agarose gel electrophoresis. A 20 μl aliquot of each amplified product was used for pyrosequencing, which was carried out on the PyroMark Q24 instrument (Qiagen) at the Australian Genome Research Facility (AGRF, Perth, AU) using the appropriate sequence primers for each gene. Assay setup, sequence run, and analysis were performed with PyroMark Q24 Software [19]. No-template negative controls, and SiHa cell line was used as a positive methylation control. Briefly, each PCR/pyrosequencing run had an established bisulphite modified SiHa cell line that had been previously validated for methylation analysis and a new SiHa cell line (bisulphite modified at the same time as the samples). This approach controlled for variation in the bisulphite modification procedure between assays and variation in methylation analysis between runs (reproducibility of % of methylation of the positive control for each gene) [17].

**Table 1:** Primers used for pyrosequencing.

Primer Name	Sequence 5’---- 3’	Size (bp)	Position in gene	CpG sites	Annealing Temp (°)	Reference
Amplification *EPB41L3* F	GGGGGATTTGTGTAAATTGG	83	376 to 458	6	54	[18]
Amplification *EPB41L3* R (Bio)	(Bio)- ACCTAAAAACCTCCCTAAAATC					
Sequencing *EPB41L3* s	GGGATTTGTGTAAATTGG					
Amplification *TERT* F (Bio)	(Bio)-GAGGGGTTGGGAGGGTT	106	−144 to −249	10	56	[18]
Amplification *TERT* R	TCCTACCCCTTCACCTTCCAA					
Sequencing *TERT* s	CCTTCACCTTCCAACT					
Amplification *FAM19A4* F	ATTAAATTAAGTAAGGGATTTGTG	152	548 to 700	5	50	New primers designed
Amplification *FAM19A4* R (Bio)	(Bio)-AACTTCAACACAAAAAAATTAAAC					
Sequencing *FAM19A4* F s	AGTAAGGGATTTGTGAGGTGG					

*EPB41L3* and *hTERT* primers directed to the promoter region, according Vasiljević et al. [18]. *FAM19A4* New primers designed to the proximal exonic region.

Reproducibility of the assays were performed utilising dilution series of SiHa cell line by triplicate in intra and inter assays and by using a training panel of samples as reported previously [14,17,18].

### 2.6 Data and statistical analysis

For pyrosequencing we calculated the percentage of methylation at individual CpG-sites [19]. Percentage of median DNA methylation for each individual CpG-site [*EPB41L3* (CpG-sites 1–6), *hTERT* (CpG-sites 1–10) and *FAM19A4* (CpG-sites 1–5)], for each disease grade [SCC, HSIL, LSIL and normal] was compared using two non-parametric comparative analyses, Wilcoxon test for between groups and Kruskal-Wallis for overall analysis, which was visualised using box and whisker plots [17]. Area under the curve (AUC) was used to assess the ability of the methylated genes at individual CpG-sites and by using the average of all CpG-sites of each gene to distinguish HSIL and SCC from normal/LSIL samples. The optimal cut-off point for each individual CpG-site was calculated by using the maximum sum of sensitivity and specificity as described previously [20]. We assessed the potential of different models in detecting HSIL or worse with respect to their sensitivity [number of correct positives (i.e. positive for at least one marker)/number of reference assay positives] and specificity (number of correct negatives on all markers/number of reference assay negatives).

Triage strategies used were (I) individual analysis of methylation at specific CpG-sites for each gene, (II) combination of two or more individual CpG-sites (III) Individual or combined methylation analysis at individual CpG-sites and adding HPV16/18 typing (IV) Individual or combined methylation analysis at individual CpG-sites and adding extended genotyping (HPV 16/18/31/33/45/52/58. Percentage of median DNA methylation for each individual CpG-site between cervical and vaginal specimens was also compared using Wilcoxon signed rank test and visualised by using box and whisker plots. The results were analysed by using XLSTAT, the statistical platform R studio (v4.0.1) and programs ggplots2 (v3.3.2), ggpubr (v0.4), pROC (v1.16.2) and cutpointr (v1.1.1) [20,21].

### 2.7 Ethical considerations

Approval of the trial and biomarker analysis was provided by the Medical Research Advisory Committee (MRAC) of the Papua New Guinea National Department of Health (approval number 17.36), the Institutional Review Board of the Papua New Guinea Institute of Medical Research (IRB 1712), and the Human Research Ethics Committee (HREC) of UNSW Australia (approval number HC17631). Written informed consent was obtained from all participants prior to enrolment.

## 3 Results

### 3.1 DNA methylation at individual CpG-sites of *EPB41L3*, *FAM19A4* and *hTERT* genes and lesion grade

We performed the analysis of DNA methylation at individual CpG-sites of *EPB41L3, hTERT* and *FAM19A4* genes in 44 hrHPV+ clinician and self-collected vaginal samples [23 cases (19 HSIL and 4 SCC 8) and 21 normal/LSIL (17 normal LBC and 4 LSIL)]. Of these samples, 42/44 (95.4%) clinician-collected cervical samples were successfully amplified for *EPB41L3* and *hTERT* (1 normal and 1 HSIL did not amplify) and 40/44 (90.9%) for *FAM19A4* (2 normal and 2 HSIL did not amplify). All the 44 self-collected vaginal samples produced an amplicon for the three genes, and thus were considered optimal for pyrosequencing.

#### 3.1.1 EPB41L3

In clinician-collected samples, we observed an increasing of DNA methylation with increasing of lesion grade at all six individual CpG-sites analysed of *EPB41L3* gene (Fig. 1). DNA methylation at each CpG site (CpG-sites 1–6) was significantly higher in HSIL than in normal/LSIL samples, and in cervical cancer compared to normal cytology (Fig. 1, top row).

**Fig. 1:**
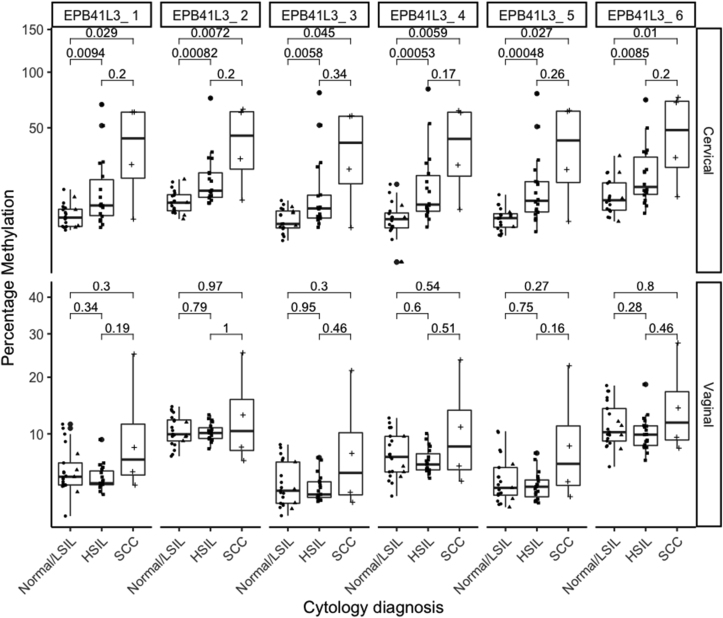
Percentage of DNA methylation at individual CpG-sites of *EPB41L3* gene according to cytology diagnosis in hrHPV+ women. Clinician-collected cervical samples (Cervical) are presented in the top panel, and self-collected vaginal samples (Vaginal) in the bottom panel. Comparisons were assessed by the nonparametric Wilcoxon test, with whiskers corresponding to the first and third quartiles (the 25th and 75th percentiles). Significance values p ≤ 0.05.

In self-collected samples, there was an increased level of methylation in women with cervical cancer compared to women with normal cytology at some individual CpG-sites (CpG-site 1, 3 and 5), but the association was not statistically significant (Fig. 1, lower row).

#### 3.1.2 hTERT

In clinician-collected samples we observed an increase of *hTERT* DNA methylation at some specific CpG-sites in women with SCC compared to women with normal cytology (CpG-site 4, p = 0.013) and in women with SCC compared to women with HSIL (CpG-site 4, p = 0.033 and CpG-site 7, p = 0.014) Fig. 2, top row.

**Fig. 2:**
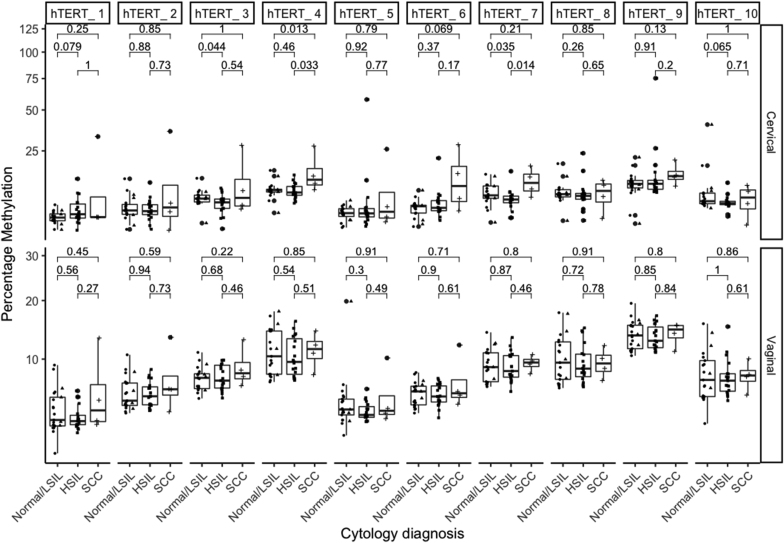
Percentage of DNA methylation at individual CpG-sites of *hTERT* gene according to cytology diagnosis in hrHPV+ women. Clinician-collected cervical samples (Cervical) are presented in the top panel and self-collected vaginal samples (Vaginal) in the lower panel. Comparisons were assessed by the nonparametric Wilcoxon test, with whiskers corresponding to the first and third quartiles (the 25th and 75th percentiles). Significance values p ≤ 0.05.

In self-collected samples, there was not a clear association of DNA methylation of *hTERT* with lesion grade (Fig. 2, lower row).

#### 3.1.3 FAM19A4

In clinician-collected samples, methylation at CpG-sites 1, 2 and 3 of *FAM19A4* showed a decrease in the levels of methylation in cancer samples compared to normal samples, although not statistically significant (p = 0.097, p = 0.096 and p = 0.077 respectively) (Fig. 3).

**Fig. 3:**
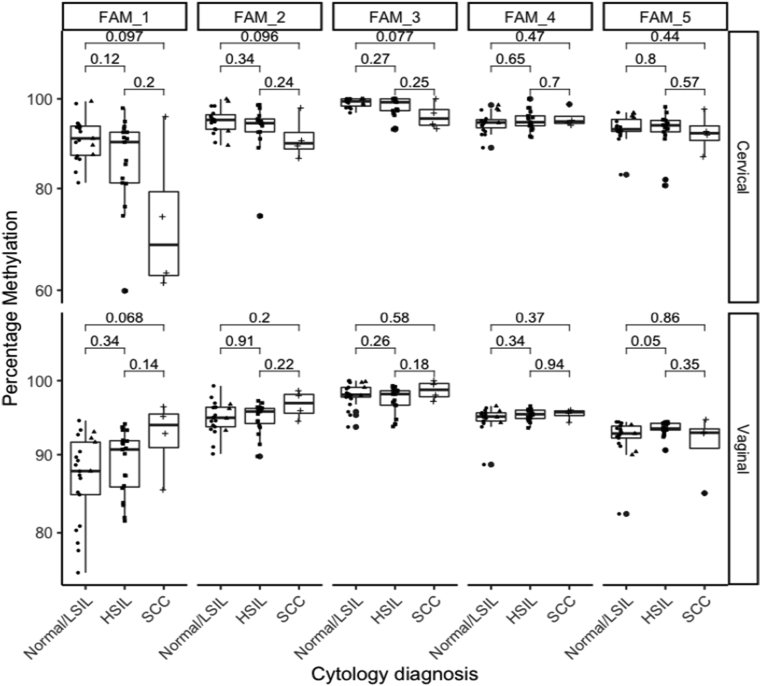
Percentage of DNA methylation at individual CpG-sites of *FAM19A4* gene according to cytological diagnosis in hrHPV+ women. Clinician-collected cervical samples (Cervical) are presented in the top panel, and self-collected vaginal (Vaginal) samples in the bottom panel. Comparisons were assessed by the nonparametric Wilcoxon test, with whiskers corresponding to the first and third quartiles (the 25th and 75th percentiles). Significance values p ≤ 0.05.

In self–collected samples, methylation at CpG-site 1 of *FAM19A4* showed an increase in the levels of methylation in cancer samples compared to normal samples (p = 0.068) and methylation at CpG-site 5 showed an increase in the levels of methylation in women with HSIL compared to women with normal samples (p = 0.05).

### 3.2 AUC of DNA methylation markers for HSIL, HSIL+ and SCC

**Fig. 4:**
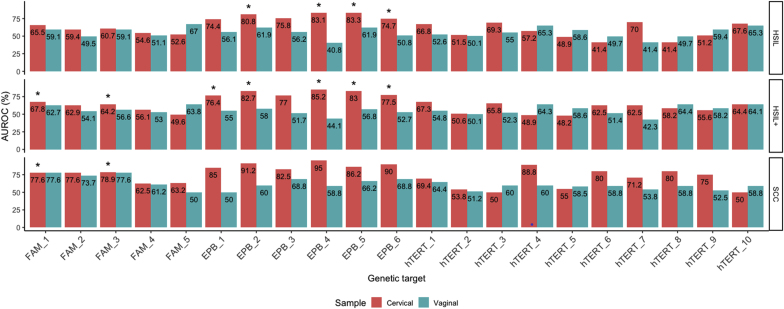
Area under the curve values of *FAM19A4* (CpG-sites 1–5)*, EPB41L3* (CpG-sites 1–6) and *hTERT* (CpG-sites 1–10) methylation for distinguishing HSIL, HSIL+ and SCC from normal/LSIL, stratified by sample type Orange bars and green bars represent clinician-collected cervical and self-collected vaginal samples respectively. HSIL, in the top panel, HSIL+ in the middle panel and SCC in the bottom panel. The p values in the figure represent p < 0.05 = *, p < 0.01 = **, p < 0.005 = ***, p < 0.001 = ****. (For interpretation of the references to color in this figure legend, the reader is referred to the Web version of this article.)

AUC analysis showed that in clinician-collected samples*, EPB41L3* was the best methylation marker to distinguish HSIL and SCC (from normal/LSIL). *EPB41L3* methylation distinguished HSIL from normal/LSIL at CpG-sites 2 (AUC value 0.808), 4 (0.831) and 5 (0.833), HSIL+ from normal/LSIL at CpG-sites 1 (0.764), 2 (0.827), 4 (0.852) and 5 (0.830), and SCC from normal/LSIL at CpG-sites 2 (0.912), 4 (0.920) and 6 (0.900). *EPB41L3* methylation distinguished HSIL and HSIL+ from normal/LSIL with significant p-values (p < 0.05) at the respective CpG-sites, while the p-values for SCC from normal/LSIL were not significant (Fig. 4). *hTERT* methylation distinguished HSIL from normal/LSIL at CpG-sites 7 (AUC value 0.700) and 3 (0.693), HSIL+ from normal/LSIL at CpG-sites 1 (0.673) and 3 (0.658) and SCC from normal/LSIL at CpG-sites 4 (0.880), 6 (0.800) and 8 (0.800). However, the p-values were not significant. *FAM19A4* methylation showed a lower performance for detecting HSIL. DNA methylation at CpG site 1 distinguished HSIL from normal/LSIL (AUC value 0.655), HSIL+ from normal/LSIL at CpG-sites 1 (0.678) and 3 (0.642) and SCC from normal/LSIL at CpG-sites 1, and 3 (AUC values of 0.776, and 0.789, respectively). *FAM19A4* methylation distinguished HSIL+ and SCC from normal/LSIL with significant p-values (p < 0.05) at CpG sites 1 and 3.

In self-collected samples, *FAM19A4* was the best methylation marker to stratify disease grade. *FAM19A4* methylation distinguished HSIL from normal/LSIL at CpG-site 5 (AUC value 0.670), and HSIL+ from normal/LSIL at CpG-site 1 (0.627) and CpG site 5 (0.638) and SCC from normal/LSIL at CpG-sites 1 (0.776), 2 (0.737) and 3 (0.776). For *EPB41L3,* CpG-site 2 distinguished HSIL from normal/LSIL with an AUC of 0.619, and methylation at CpG-sites 3 and 6 distinguished SCC from normal/LSIL with an AUC of 0.688 each.

For *hTERT,* methylation at CpG-site 4 and site 10 were the best markers to distinguish HSIL and HSIL+ from normal/LSIL and CpG-sites 1, 3 and 4 distinguished SCC from normal/LSIL (Fig. 4). However, the p-values for *FAM19A4, EPB41L3* and *hTERT* at the respective CpG sites were not significant.

Global methylation analysis for each gene (the average of all the CpG-sites) performed best for *EPB41L3* using clinical-collected samples and *FAM19A4* in self-collected samples (Fig. 4).

### 3.3 Diagnostic performance of DNA methylation markers in the detection of HSIL+

In clinician-collected samples methylation at CpG-site 4 of *EPB41L3* showed the highest individual performance for HSIL+ detection, with a sensitivity of 86.4% and specificity of 70%. Combined detection of methylation at CpG-site 2 and 4 of *EPB41L3* improved the diagnostic performance with a sensitivity of 95.5% and specificity of 60%; when these two CpG-sites were further combined with methylation at CpG-site 1 of *FAM19A4*, the best diagnostic performance was reached with a sensitivity of 100% and specificity of 63.2% (Table 2).

**Table 2:** Diagnostic performance of methylation markers at individual CpG-sites in the detection of HSIL + on clinician-collected samples and self-collected samples.

Sample type	Gene and CpG-sites^a^	Best cut off (% methylation)	Sensitivity % (95 % CI)	Specificity % (95 % CI)
*Cervical, clinician-collected*	*EPB41L3* CpG site 1	5	81.8 (59.7–94.8)	40.0 (19.1–63.9)
	*EPB41L3 CpG-site 2*	10.5	86.3 (65.1–97.1)	65.0 (40.8–84.6)
	*EPB41L3 CpG-site 3*	5	81.8 (59.7–94.8)	55.0 (31.5–77.1)
	*EPB41L3 CpG-site 4*	6	86.4 (65.1–97.1)	70.0 (45.7–88.1)
	*EPB41L3 CpG-site 5*	6	81.8 (59.1–94.8)	60.0 (36.1–88.9)
	*EPB41L3 CpG-site 6*	10.5	86.4 (65.1–97.1)	50.0 (27.2–72.8)
	*EPB41L3 CpG-site 2/site 4*	10.5/6	95.5 (77.2–99.9)	60.0 (36.1–88.9)
	*EPB41L3 CpG-site 2/site 5*	10.5/6	90.9 (70.8–98.9)	50.0 (27.2–72.8)
	*EPB41L3 CpG-site 4/site 5*	6/6	90.9 (70.8–98.9)	50.0 (27.2–72.8)
	*hTERT CpG-site 4*	9.2	50.0 (28.2–71.8)	70.0 (45.7–88.1)
	*FAM19A4 CpG-site 1*	<86	52.4 (29.8–74.3)	89.5 (66.9–98.7)
	*EPB41L3 CpG-site 4/FAM19A4 site 1*	6/<86	90.5 (69.6–98.8)	68.4 (43.5–87.4)
	*EPB41L3 CpG-site 2/4/FAM19A4 site 1*	10.5/6/<86	100 (83.9–100)	63.2 (38.4–83.7)
	*EPB41L3 CpG-site 4/hTERT site 4*	6/9.2	90.9(70.8–98.9)	60.0 (36.1–80.9)
	*EPB41L3 CpG-site 2/*4/hTERT *site 4*	10.5/6/9.2	95.5 (77.2–99.9)	50.0 (27.2–72.8)
	b*Global EPB41L1 CpG-site 1/2/3*	Mean site 1/2/3 (7.5)	77.3 (54.6–92.2)	60.0 (36.1–80.9)
	c*Global EPB41L3 CpG-site 1-6*	Mean site 1/2/3/4/5/6 (7.5)	81.8 (59.7–94.8)	65.0 (40.8–84.6)
	d*Global FAM19A4 CpG-site 1-5*	Mean site 1/2/3/4/5 (<93)	52.4 (29.8–74.3)	84.2 (60.4–96.6)
	e*Global hTERT CpG-site 1-10*	Mean sites 1–10 (7)	54.6 (32.2–75.6)	60.0 (36.1–80.9)
*Vaginal, self-collected*	*EPB41L3 CpG-site 2*	9	82.6 (61.2–95.1)	28.6 (11.3–52.2)
	*EPB41L3 CpG-site 3*	3	65.2(42.7–83.6)	38.1 (18.1–61.6)
	*EPB41L3 CpG-site 2/site 3*	9/3	91.3(72.0–98.9)	23.8 (8.2–47.2)
	*FAM19A4 CpG-site 1*	90	60.9 (38.6–80.3)	66.7 (40.0–85.4)
	*FAM19A4 CpG-site 5*	92.5	87.0 (66.4–97.2)	42.9 (21.8–66.0)
	*FAM19A4 CpG-site 1/site 5*	90/92.5	95.7 (78.1–99.8)	28.6 (11.3–52.2)
	*EPB41L3 CpG-site 3/FAM19A4 site 1*	3/90	91.3 (72.0.-98.9)	23.8 (8.2–47.2)
	*EPB41L3 CpG-site 3/FAM19A4 site 5*	3/92.5	100 (85.2–100)	9.5 (1.2–30.4)
	*hTERT CpG-site 4*	8	73.9 (51.6–89.8)	33.3 (14.6–57.0)
	*hTERT CpG-site 10*	5.5	91.3 (72.0–98.9)	33.3 (14.6–57.0)
	*EPB41L3 CpG-site* 3/hTERT *site 4*	3/8	95.7 (78.1–99.9)	19.1 (5.5–41.9)
	*EPB41L3 CpG-site3/hTERT site 10*	3/5.5	100 (85.2–100)	14.3 (3.0–36.3)
	*FAM19A4 CpG-site1/hTERT site 4*	90/8	82.6 (61.2–95.1)	23.8 (8.2–47.2)
	*FAM19A4 CpG-site* 1/hTERT *site 10*	90/5.5	95.7 (78.1–99.9)	23.8 (8.2–47.2)
	b*Global EPB41L3 CpG-site1/2/3*	Mean site 1/2/3 (5.5)	69.6 (47.1–86.8)	33.3 (14.6–57.0)
	c*Global EPB41L3 CpG-site 1-6*	Mean site 1/2/3/4/5/6 (6)	65.2 (42.7–83.6)	38.1 (18.1–61.6)
	d*Global FAM19A4 CpG-site 1-5*	Mean site 1/2/3/4/5 (93.5)	69.6 (41.1–86.8)	42.9 (21.8–66.0)
	e*Global hTERT CpG-site 1-10*	Mean site 1–10 (6.5)	73.9 (51.6–89.8)	28.6 (11.3–52.2)

High-light in grey individual CpG-sites or combination of different CpG-sites with the best diagnostic performance for detection of HSIL+.

a Positive for at least one of the indicated CpG-sites.

b Global *EPB41L*3 CpG site 1/CpG site 2/CpG site 3 is equivalent to the three CpG-sites of *EPB41L3* (438, 427 and 425) studied by Lorincz group and others. There is the S5 classifier methylation assay that include, the mean % of *EPB41L3* methylation of these three CpG-sites, the mean % of methylation of HPV16-L1: 6367, 6389; HPV18-L2: 4256, 4261, 4265, 4269, 4275, 4281; HPV31-L1: 6352 and 6364 and HPV33-L2: 5557, 5560, 5566) and the proportion of CpGs methylated in HPV16-L2 sites: 4238, 4259, 4275 for diagnostic detection of CIN2+.

c Global *EPB41L*3 gene: mean % of methylation of CpG-sites 1/2/3/4/5/6.

d Global *FAM19A4* gene: mean % of methylation of CpG-sites 1/2/3/4/5.

e Global *hTER*T gene: mean % of methylation of CpG-sites 1/2/3/4/5/6/7/8/9/10.

In self-collected samples, methylation at CpG-site 5 of *FAM19A4* showed the best individual diagnostic performance with a sensitivity of 87% and specificity of 42.9% (Table 2). Combined detection of at least two methylation markers improved the sensitivity (values between 82.6% and 100%), but with a low specificity (values between 4.5% and 28.3%) (Table 2).

### 3.4 Diagnostic performance of DNA methylation at specific CpG-sites combined with HPV infection for the detection of HSIL+

For clinician-collected samples, adding HPV16/18 detection to methylation at CpG-site 4 of *EPB41L3* increased sensitivity from 86.4% to 100%, and reduced specificity from 70.0% to 55% for detection of HSIL+. Combination of methylation at CpG-site 2 and 4 of *EPB41L3* and adding HPV16/18 detection increased sensitivity from 95.5% to 100% but reduced specificity from 60% to 50% (Table 3).

**Table 3:** Performance of HPV genotyping combined with methylation at specific CpG-sites for the detection of HSIL+.

Sample type	Combined HPV/methylation detection for decision making			Sensitivity % (95 % CI)	Specificity % (95 % CI)
	HPV genotype^a^	Host Gene^b^	Best cut off (% methylation)		
*Cervical, clinician-collected*	16	NA	NA	52.2 (30.6–73.2)	81.0 (58.1–94.6)
	16,18	NA	NA	60.9 (38.5–80.3)	81.0 (58.1–94.6)
	16,18,31,33,45,52,58	NA	NA	95.7 (78.1–99.9)	33.3 (14.6–56.9)
	16,18	*EPB41L3 CpG-site 2*	10.5	90.9 (70.8–98.9)	50.0 (27.2–72.8)
	16,18,31,33,45,52,58	*EPB41L3 CpG-site 2*	10.5	100 (84.6–100)	25.0 (8.7–49.1)
	16,18	*EPB41L3 CpG-site 4*	6.0	100 (84.6–100)	55.0 (31.5–76.9)
	16,18,31,33,45,52,58	*EPB41L3 CpG-site 4*	6.0	100 (84.6–100)	30.0 (11.9–54.3)
	16,18	*EPB41L3 CpG-site 5*	6.0	90.9 (70.8–98.9)	50.0 (27.2–72.8)
	16,18,31,33,45,52,58	*EPB41L3 CpG-site 5*	6.0	100 (84.6–100)	25.0 (8.7–49.1)
	16,18	*EPB41L3 CpG-site 2/4*	10.5/6.0	100 (84.6–100)	50.0 (27.2–72.8)
	16,18,31,33,45,52,58	*EPB41L3 CpG-site 2/4*	10.5/6.0	100 (84.6–100)	25.0 (8.7–49.0)
	16,18	*EPB41L3 CpG-site2/5*	10.5/6.0	95.5 (77.2–99.9)	40.0 (19.1–64.0)
	16,18,31,33,45,52,58	*EPB41L3 CpG-site 2/5*	10.5/6.0	100 (84.6–100)	20.0 (5.7–43.7)
	16,18	*EPB41L3 CpG-site 4/5*	6.0/6.0	100 (84.6–100)	45.0 (23.1–68.5)
	16,18,31,33,45,52,58	*EPB41L3 CpG-site 4/5*	6.0/6.0	100 (84.6–100)	30.0 (11.9–54.3)
	16,18	*EPB41L3 CpG-site 1/2/3*	5.0/10.5/5	95.5 (77.2–99.9)	30.0 (11.9–54.3)
	16,18,31,33,45,52,58	*EPB41L3 CpG-site 1/2/3*	5.0/10.5/5	100 (84.6–100)	15.0 (3.2–37.9)
	16,18	*Global EPB41L3 CpG-site 1/2/3*	Mean1/2/3(7)	86.4 (65.1–97.1)	45.0 (23.1–68.5)
	16,18,31,33,45,52,58	*Global EPB41L3 CpG- site 1/2/3*	Mean1/2/3(7)	95.5 (77.2–99.9)	25.0 (8.7–49.1)
	16,18	*FAM19A4 CpG-site 1*	<86	76.2 (52.8–91.8)	68.4 (43.5–87.4)
	16,18,31,33,45,52,58	*FAM19A4 CpG-site 1*	<86	95.2 (76.2–99.9)	26.3 (9.2–51.2)
	16,18	*hTERT CpG-site 4*	9.2	72.7 (49.8–89.3)	55.0 (31.5–76.9)
	16,18,31,33,45,52,58	*hTERT CpG-site 4*	9.2	100 (84.6–100)	35.0 (15.4–59.2)
*Vaginal, self-collected*					
	16,18	NA	NA	60.9 (38.5–80.3)	81.0 (58.1–94.6)
	16,18,31,33,45,52,58	NA	NA	95.7 (78.1–99.9)	33.3 (14.6–56.9)
	16,18	*EPB41L3 CpG-site 2*	9	87.0 (66.4–97.2)	23.8 (8.2–47.2)
	16,18,31,33,45,52,58	*EPB41L3 CpG-site 2*	9	100 (85.2–100)	4.8 (0.1–23.8)
	16,18	*FAM19A4 CpG-site 1*	90	82.6 (61.2–95.1)	61.9 (38.4–81.9)
	16,18,31,33,45,52,58	*FAM19A4 CpG-site 1*	90	100 (85.2–100)	23.8 (8.2–47.2)
	16,18	*FAM19A4 CpG-site 5*	92.5	95.7 (78.1–99.9)	28.6 (11.3–52.2)
	16,18,31,33,45,52,58	*FAM19A4 CpG-site 5*	92.5	100 (85.2–100)	9.5 (1.2–30.4)
	16,18	*hTERT CpG-site 4*	8.0	91.3 (72.0–98.9)	23.8 (8.2–47.2)
	16,18,31,33,45,52,58	*hTERT CpG-site 4*	8.0	100 (85.2–100)	14.3 (3.1–36.3)
	16,18	*hTERT CpG-site 10*	5.5	95.7 (78.1–99.9)	23.8 (8.2–47.2
	16,18,31,33,45,52,58	*hTERT CpG-site 10*	5.5	100 (85.2–100)	14.3 (3.1–36.3)

High-light CpG-sites and HPV typing with the best diagnostic performance for detection of HSIL or worse.

a Detection of at least one of the indicated HPV genotypes.

b Positive for at least one of the indicated CpG-sites.

Combination of any methylation marker with extended genotyping (HPV16/18/31/33/45/52/58), showed a sensitivity of 100% at the expense of a low specificity (values between 15.0% and 35.0%).

In self-collected samples, adding HPV16/18 detection to methylation at CpG-site 1 of *FAM19A4* showed the best diagnostic performance with a sensitivity of 82.6% and specificity of 61.9%. Methylation at CpG-site 5 of *EPB41L3* plus HPV16/18 detection also showed a high sensitivity of 95.7% but at expenses of a low specificity of 28.6%. Combination of any methylation marker with extended genotyping (HPV16/18/31/33/45/52/58), showed a sensitivity of 100% at the expense of a low specificity (values between 4.8% and 23.0%).

### 3.5 Comparison of methylation between paired self-collected and clinician-collected samples

Analysis of methylation at specific CpG-sites for *EPB41L3* and *hTERT* genes was performed in 42 patients, and for *FAM19A4* in 40 patients with paired assessable samples. For women with HSIL and SCC, the levels of methylation at all CpG-sites (1–6) of *EPB41L3* in self-collected samples were lower than the levels of methylation of their paired clinician-collected samples (Supplemental Fig. S1).

In contrast, for women with HSIL and normal/LSIL the levels of methylation of *hTERT* were higher at almost all the CpG-sites in self-collected samples compared to paired clinician-collected samples (Supplemental Fig. S2). In women with SCC, there was no clear trend.

For *FAM19A4,* significant differences in methylation at individual sites were found for women with normal/LSIL samples (higher for clinician-collected samples at CpG-sites 1 and 3 compared to paired self-collected samples). However, in women with HSIL and cancer, methylation at CpG site 1 seems to be higher for self-collected samples than clinician-collected samples (Supplemental Fig. S3).

## 4 Discussion

This study analysed the clinical performance of DNA methylation at individual CpG-sites of *EPB41L3, hTERT* and *FAM19A4* for predicting HSIL+ in clinician and self-collected samples from HPV+ women from PNG, where the burden of hrHPV infection and disease are high, and the development of new triage strategies for detection of HSIL+ are urgently needed. Methylation at individual CpG-sites in clinician and self-collected allowed us to differentiate HSIL vs normal samples, and cancer vs normal samples and to define the best combination of markers, algorithms and thresholds for the detection of HSIL+. The best clinical performance for detection of HSIL+ was obtained by using *EPB41L3* site 2/4*/FAM19A4* site 1 with a sensitivity of 100% and specificity of 63.2% for clinician collected samples, and *FAM19A4* site 1 combined with HPV16/18 with a sensitivity of 82.6% and specificity of 61.9% for self-collected samples. Comparison of DNA methylation at individual CpG-sites of these genes in paired self/clinician-collected samples differed according to the sample type, lesion grade and gene analysed, generating novel information at basic, clinical and epidemiological level. Our study demonstrated the importance of performing analysis at individual CpG-sites on the promoter and proximal exonic regions of host genes, as some CpG-sites appear to be more susceptible to tumour-associated changes than others.

### 4.1 DNA methylation of *EPB41L3*

*EPB41L3* is a tumour suppressor gene that suppresses metastasis by regulating the proper arrangements of actin stress fibres and increasing cell motility associated with metastatic behaviour [22]. Hyper-methylation of the *EPB41L3* promoter down-regulates expression during tumour development in ovarian, lung, cervix, breast, prostate and oral squamous cell carcinomas [18,23,24]. In cervical disease, methylation of *EPB41L3* has been evaluated in clinical studies generally with global analysis of the percentage of methylation in the gene [25]. Increased levels of DNA methylation according to lesion grade have been observed in clinician-collected samples from high-income populations by using different techniques and analysing the gene alone or in combination with other genes [13,18,[26], [27], [28], [29]]. In LMIC, only one study has been performed using clinician collected samples, also showing increased levels of methylation according lesion grade [30]. Although we cannot perform a direct comparison of our results with other studies, when we performed global methylation analysis of the *EPB41L3* gene, our results are similar to those obtained in high-income populations [18,28,31,32]. In our exploratory setting, this gene showed a promising performance for the detection of HSIL, HSIL+ and SCC, making it a striking triage marker to validate in large scale studies in LMIC. Notably, we found individual CpG-sites yield higher performance than an averaged “global” methylation across the region. These values were also higher than those obtained by other researchers using a global approach [28,30,31], highlighting the importance of considering analysis at individual CpG-sites.

In our study analysis of methylation at individual CpG site 2/4 of *EPB41L3* increased the diagnostic performance for the detection of HSIL+ with an excellent sensitivity of 95.5% and a specificity of 60% in clinician-collected samples. However, the best diagnostic performance was observed by combining methylation of *EPB41L3* site 2/4 and *FAM19A4* site 1, showing that combination of individual CpG sites of different genes could improve the detection of HSIL+. By using any of these models, the percentage of referrals to treatment in our PNG exploratory study would theoretically have been 60–70%, lower than that of transferring all hrHPV + women under the current algorithm, thus reducing unnecessary referrals and overtreatments without affecting sensitivity.

A study analysed specific CpG sites methylation and their role in the *EPB41L3* expression in gastric cancer, finding a strong correlation between CpG hypermethylation and decreased *EPB41L3* mRNA and protein levels [33]. Recently an integrated bioinformatics approach identified that *EPB41L3* was hypermethylated and correlated with a decreased expression of *EPB41L3* mRNA in cervical cancer tissues compared with normal tissues. In addition, a lower expression of the gene was correlated with a shorter survival time [34].

In self-collected samples, the performance of *EPB41L3* methylation for detection of HSIL or cancer was lower than in clinician-collected cervical samples. A few studies, using qMSP, have analysed methylation of this gene in self-collected samples, all performed in high-income countries [26,35,36]. Two of them compared methylation levels of self-collected samples with clinician collected samples and both showed decreased methylation levels in self-collected samples when compared with clinician-collected samples, as observed in our study. Furthermore, to the best of our knowledge, our study is the first one to perform methylation analysis of the *EPB41L3* gene (global and at individual CpG-sites) in self-collected samples from a LMIC. Global methylation analysis of *EPB41L3* in these samples showed a limited sensitivity/specificity for the detection of HSIL+ but analysis of methylation at individual CpG-sites 2 and 3 increased the sensitivity for the detection of HSIL+ to 91.3% at the expense of a low specificity. Similar results were observed when we combined methylation at individual CpG-sites with HPV16/18 or extended genotyping. In summary these results show that analysis of *EPB41L3* methylation will be better to analyse in clinician collected samples in case that this gene is used for diagnosis of HSIL+ for screening purpose in this setting.

### 4.2 DNA methylation of *hTERT*

DNA methylation plays a role in deregulated *hTERT* expression and is implicated in HPV-mediated pathogenesis of cervical cancer [[37], [38], [39], [40], [41]]. Studies using clinician-collected samples from high-income populations have found increasing *hTERT* methylation with escalating lesion grade [26,37,42]. Only one study has been done in a LMIC, in cervical cancer biopsies [41].

In our study, global methylation analysis showed a similar performance for detection of HSIL+ as observed in high income countries for CIN2+ in clinician-collected samples (sensitivities 40–69% and specificities 54–88%) [18,26,35] and in self-collected samples (sensitivity of 62.9%) [43].

Analysis at individual CpG-sites found site 4 was the best marker to distinguish normal/cancer samples and CpG site 4 and 10 the best markers to distinguish normal/HSIL in clinician and self-collected samples respectively. The *hTERT* promoter region harbors binding sites for transcription factors that positively or negatively regulate *hTERT* expression. *SMAD3* is a repressor protein that binds to the *hTERT* gene promoter (from position −218 to −206 to the transcriptional initiation codon) and inhibits *hTERT* gene transcription activity [44,45]. Methylation of site 4 (position −212) could reduce the binding of *SMAD3* favouring *hTERT* transcription, but additional research at transcriptional level is required to confirm this idea. A study in colorectal samples showed that three specific CpG sites in the *hTERT* promoter region were related with increasing of *hTERT e*xpression during malignant progression of colorectal carcinoma [46]. Hence, some CpG-sites appear to be more susceptible to tumour-associated changes than others.

### 4.3 DNA methylation of *FAM19A4*

*FAM19A4* is a member of the TAFA family of five highly homologous genes that encode small, secreted proteins predominantly expressed in the brain [47]. *FAM19A4* also has been identified as a promising biomarker for cervical oncogenesis by using genome wide DNA screening [48]. Furthermore, *FAM19A4* promoter methylation analysis has been shown to predict underlying CIN3/CIN3+ [49].

In our study, we used a new set of primers for analysis of DNA methylation directed to the proximal exonic region in the gene *FAM19A4,* showing promising results for detection of HSIL+. We performed the methylation analysis globally and at individual CpG-sites by using pyrosequencing to get comprehensive information on the methylation status in this region and its possible biological, and diagnostic role in cervical disease. Although this exon region has not been analysed before, and a direct comparison is not possible, global methylation results in clinician-collected samples are similar to reports analysing the promoter region of *FAM19A4* in high income countries [49]. The performance of *FAM19A4* in combination with *miR124-2* methylation have shown to improve the sensitivity (range of reported studies from 68.2 to 86.7%) and specificity (ranging from 60.6 to 91%) for the detection of CIN3+ in these cervical specimens [9,[50], [51], [52]].

We found that some specific CpG sites were promising to distinguish HSIL and cancer from normal/LSIL and combination of *EPB41L3* sites 2/4 plus *FAM19A4* site 1 dramatically improved sensitivity to 100%, with a specificity of 63.2% for detection of HSIL+. These results warrant more studies of DNA methylation at specific CpG sites in the proximal exonic region of *FAM19A4* and its function in cervical carcinogenesis.

In self-collected samples, methylation at some specific CpG sites of *FAM19A4* also were promising to distinguish HSIL and cancer from normal/LSIL samples and combining *FAM19A4* CpG site1 and HPV16/18 detection showed the best diagnostic performance with a sensitivity and specificity values [82.6% (61.2–95.1) and 61.9% (38.4–81.9) respectively]. Combining this site 1 with extended genotyping increased sensitivity of 100% at expenses of specificity (23.8%). Despite the reduced specificity, employing either of these models would have reduced the referrals to treatment in our exploratory study compared to hrHPV typing alone, reducing overtreatment and the burden on the limited health resources.

Analysis of the promoter region of *FAM19A4* combined with other genes and/or HPV16/18 have shown a good performance for CIN3+ in self collected samples, in high income countries [53,54]. The results from these studies highlight the promising performance of *FAM19A4* methylation in this type of samples.

### 4.4 Levels of methylation at specific CpG-sites in paired samples differed according to lesion grade, type of sample and gene analysed

Analysis of methylation in paired samples showed that the levels of methylation at specific CpG sites not always follow the same pattern, reflected in the significant p value for some CpG sites and not others. Self-collected vaginal and clinician collected cervical samples are different. While in the self-collected vaginal sample, the women obtain cells from the vagina (a mid-cavity vaginal specimen) by using a swab or brush, the clinician collected cervical sample is collected by a clinician by using a Cervex-Brush Combi device which enable simultaneous collection of specific cells of the ectocervix, endocervix, and transformation zone in a single sample. Therefore, paired samples will have differences in cellular composition, cellular environment, and cells with differences in methylation levels, influenced by these factors but also related to the disease stage, gene, region and the specific CpG site studied, etc. These results have important implications for the translation of methylation findings from one sampling strategy to another and confirm the importance of defining specific thresholds and algorithms for the detection of HSIL+ lesions according to the sample type and other characteristics.

Limitations of this study include the small sample size specially on the number of SCC as these results were part of an exploratory study, leading to broad confidence intervals which restricted the statistical power of the current analysis. However, these results showed the reliability of the markers evaluated and allowed us to define thresholds and choose the best methylation markers and CpG-sites to be evaluated in all hrHPV+ women that participated in the PNG trial. Diagnosis was based on LBC HSIL rather than a histological end-point. Although histology is the best end point in high-income countries, in many LMIC and remote settings it is not feasible to perform colposcopy and histological diagnosis which can lead to disease misclassiﬁcation. We used some strategies to decrease disease misclassiﬁcation: HPV detection was used as primary screening tool, all slides were assessed by two independent experienced cytologists working at VCS in Melbourne, dual p16/Ki-67 immuno-staining was performed to resolve disagreements and diagnosis based in LBC HSIL have shown to be highly predictive of underlying histological disease in some settings [16].

This study is innovative as for the first time, individual CpG sites of different genes (alone or in combination) were proposed as candidates for detection of HSIL+ showing increased sensitivity and specificity in the detection of HSIL+ in both, clinician and self-collected samples, supporting this strategy to be validated in large scale studies. Also, analyses of the proximal exon region of *FAM19A4* gene, not explored previously, allowed us to identify methylation at one specific CpG site that showed very promising results for detection of HSIL+ when combined with other markers, these results show that this region is also important for the control of expression of *FAM19A4* and in cervical carcinogenesis.

In conclusion, individual CpG methylation of *EPB41L3* (promoter region) and *FAM19A4* (proximal exonic region) highlighted in this study had high performance for detection of HSIL+ in clinician and self-collected samples and warrant further evaluation. The introduction of triage assays targeting methylation at individual CpG-sites could help to reduce overtreatment rates, loss of follow-up associated with repeated clinic visits, and improvement of care in LMIC. In PNG where POC hrHPV detection is used, overtreatment could be reduced by performing methylation analysis from self-collected or clinician-collected samples prior to referring the hrHPV+ woman to ablative treatment. Large-scale implementation studies are underway across multiple LMIC settings that will allow us to confirm findings presented in current paper.

## Disclosure of potential conflicts of interest

AJV, JG, JB, GMM, PJT, SGB, JMK have received subsidized test kits for research from Cepheid. MS, JMLB, GT, DH have received donated test kits for research from Roche, Abbott, Seegene, Cepheid, Aus Diagnostics and Becton Dickinson. AJV and MS jointly lead the *Elimination of Cervical Cancer in the* 10.13039/100007159*Western* *Pacific (ECCWP)* program with philanthropic funding support from the 10.13039/501100016056Minderoo Foundation and the Frazer Family Foundation; and equipment, tests and consumables donated by 10.13039/100017037Cepheid for HPV-based cervical screening in Papua New Guinea and Vanuatu. SMG is a member of the 10.13039/100008086Global Advisory Board HPV 10.13039/100004334Merck, and has led investigator-initiated grants from 10.13039/100004334Merck on HPV in young women. MM, DAM, SP, PB, RH, ZK, GLM, declare no conflicting interests.

## Data statement

The data are not publicly available due to confidentiality and ethical considerations. Deidentified data are available from the authors upon reasonable request and subject to approval by the ethics committees overseeing the study.

## Grant support

This work was funded through the 10.13039/501100000925National Health and Medical Research Council, Australia (10.13039/501100000925NHMRC), grants 1,013,209 and 1104938) (AJV), Government of Papua New Guinea (ICRAS 297/1), the 10.13039/501100000925NHMRC Centre for Research Excellence in Cervical Cancer Control APP1135172 (JMLB, MS, AJV) and 10.13039/501100000925NHMRC Investigator Grant APP1197951 (SMG).

## CRediT authorship contribution statement

**Monica Molano:** Writing – original draft, Visualization, Software, Methodology, Investigation, Data curation, Conceptualization. **Dorothy A. Machalek:** Writing – review & editing, Visualization, Supervision, Investigation, Funding acquisition. **Samuel Phillips:** Writing – review & editing, Software, Methodology, Data curation. **Grace Tan:** Writing – review & editing, Project administration, Methodology, Investigation. **Suzanne M. Garland:** Writing – review & editing, Supervision, Project administration, Investigation, Funding acquisition. **David Hawkes:** Writing – review & editing, Methodology, Investigation. **Prisha Balgovind:** Writing – review & editing, Methodology, Investigation. **Reza Haqshenas:** Writing – review & editing, Methodology, Investigation. **Steve G. Badman:** Writing – review & editing, Project administration, Methodology, Conceptualization. **John Bolnga:** Writing – review & editing, Methodology. **Josephine Gabuzzi:** Writing – review & editing, Project administration. **Zure Kombati:** Writing – review & editing, Project administration, Investigation. **Gloria M. Munnull:** Writing – review & editing, Project administration, Investigation. **Julia ML. Brotherton:** Writing – review & editing, Funding acquisition, Conceptualization. **Marion Saville:** Writing – review & editing, Funding acquisition, Conceptualization. **John M. Kaldor:** Writing – review & editing, Funding acquisition, Conceptualization. **Pamela J. Toliman:** Writing – review & editing. **Andrew J. Vallely:** Writing – review & editing, Supervision, Project administration, Methodology, Funding acquisition, Formal analysis, Conceptualization. **Gerald L. Murray:** Writing – review & editing, Visualization, Supervision, Project administration, Investigation, Funding acquisition.

## Declaration of competing interest

The authors declare the following financial interests/personal relationships which may be considered as potential competing interests.

AJV, JG, JB, GMM, PJT, SGB, JMK have received subsidized test kits for research from Cepheid. MS, JMLB, GT, DH have received donated test kits for research from Roche, Abbott, Seegene, Cepheid, Aus Diagnostics and Becton Dickinson. AJV and MS jointly lead the *Elimination of Cervical Cancer in the* 10.13039/100007159*Western* *Pacific (ECCWP)* program with philanthropic funding support from the 10.13039/501100016056Minderoo Foundation and the Frazer Family Foundation; and equipment, tests and consumables donated by 10.13039/100017037Cepheid for HPV-based cervical screening in Papua New Guinea and Vanuatu. SMG is a member of the 10.13039/100008086Global Advisory Board HPV 10.13039/100004334Merck, and has led investigator-initiated grants from 10.13039/100004334Merck on HPV in young women. All other authors report no potential conflicts.

## Figures and Tables

**figs1:**
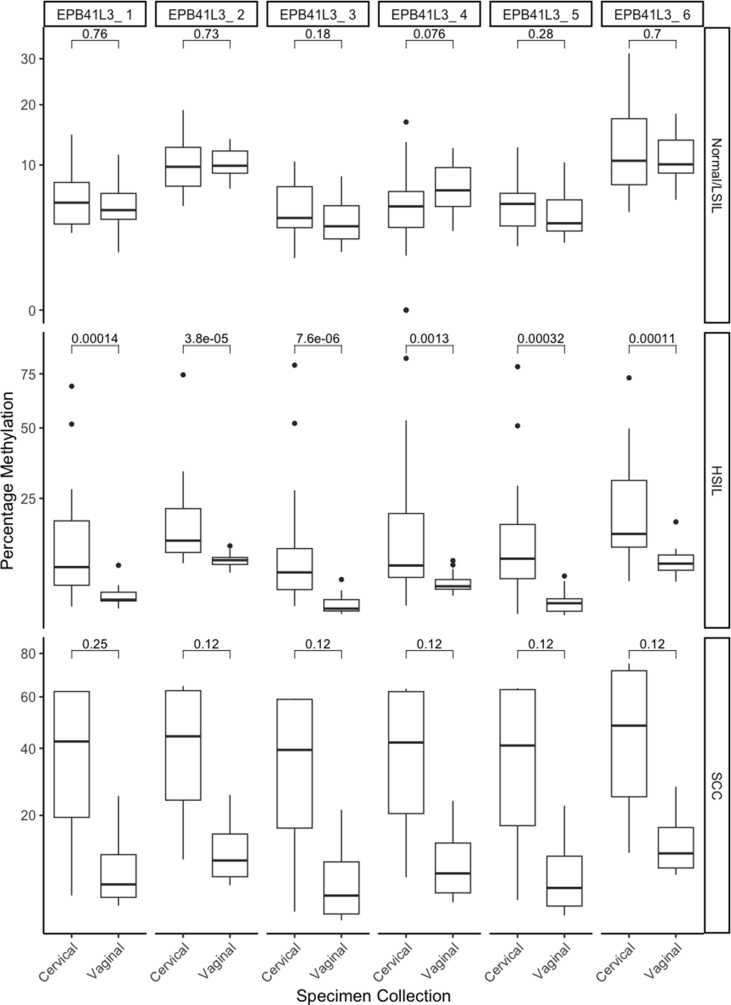


**figs2:**
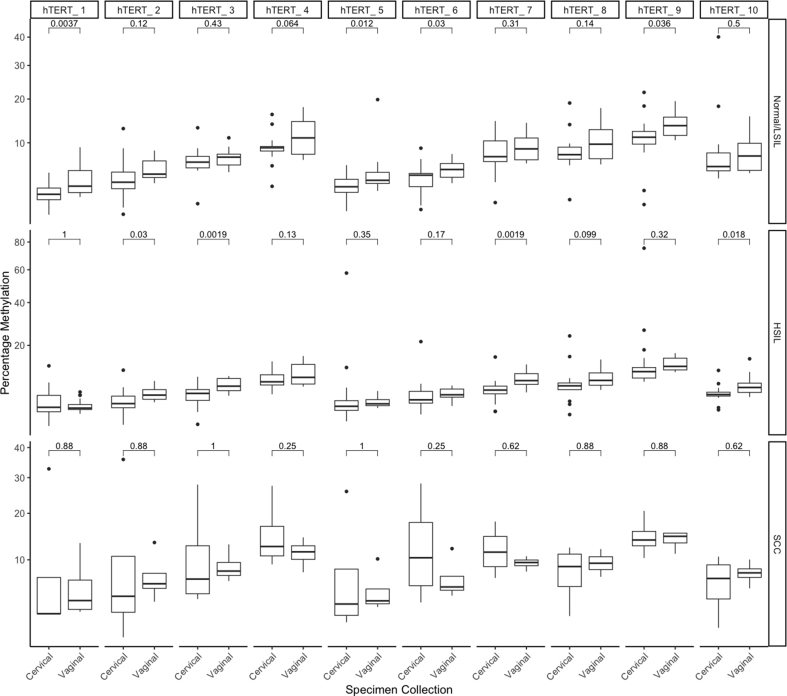


**figs3:**
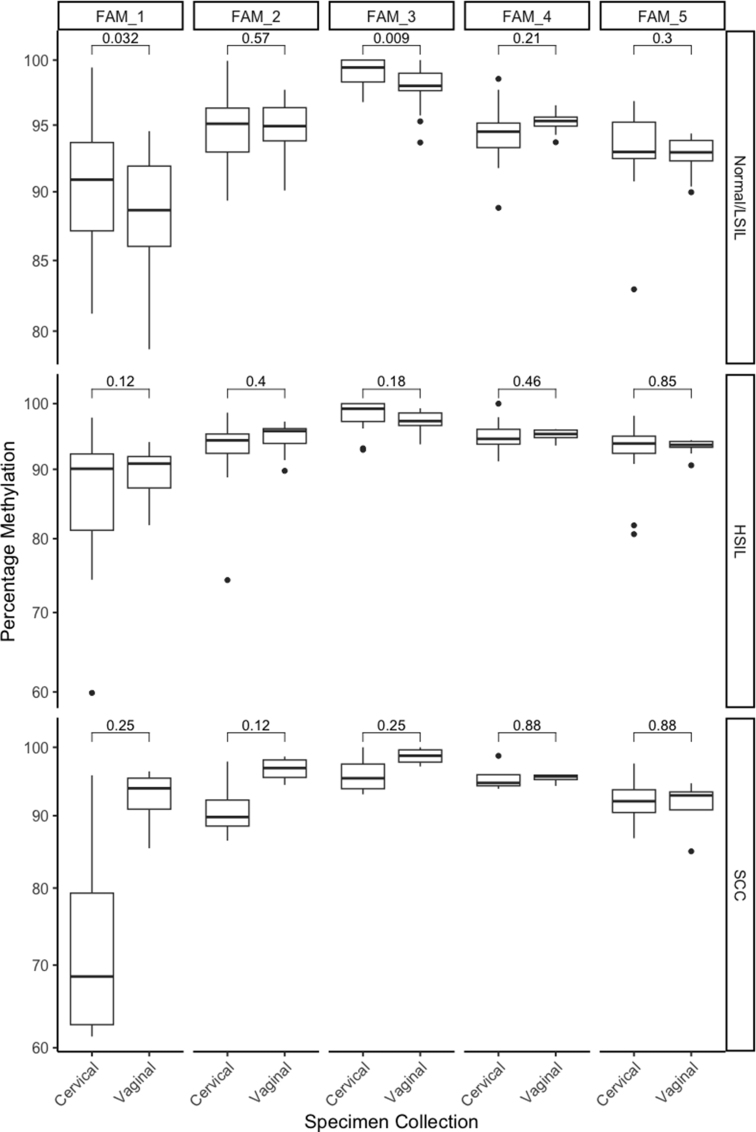


## Data Availability

The data that has been used is confidential.

## References

[1] Gravitt P.E., Silver M.I., Hussey H.M., Arrossi S., Huchko M., Jeronimo J. (2021). Achieving equity in cervical cancer screening in low- and middle-income countries (LMICs): Strengthening health systems using a systems thinking approach. Prev. Med..

[2] Cuschieri K., Ronco G., Lorincz A., Smith L., Ogilvie G., Mirabello L. (2018). Eurogin roadmap 2017: triage strategies for the management of HPV-positive women in cervical screening programs. Int. J. Cancer.

[3] Polman N.J., de Haan Y., Veldhuijzen N.J., Heideman D.A.M., de Vet H.C.W., Meijer C.J.L.M. (2019). Experience with HPV self-sampling and clinician-based sampling in women attending routine cervical screening in The Netherlands. Prev. Med..

[4] Maver P.J., Poljak M. (2020). Primary HPV-based cervical cancer screening in Europe: implementation status, challenges, and future plans. Clin. Microbiol. Infect..

[5] Machalek D.A., Roberts J.M., Garland S.M., Thurloe J., Richards A., Chambers I. (2019). Routine cervical screening by primary HPV testing: early findings in the renewed National Cervical Screening Program. Med. J. Aust..

[6] World Health Organization (2019).

[7] Ramírez A.T., Sánchez G.I., Nedjai B., Agudelo M.C., Brentnall A.R., Cuschieri K. (2021). Effective methylation triage of HPV positive women with abnormal cytology in a middle-income country. Int. J. Cancer.

[8] Wentzensen N., Schiffman M., Palmer T., Arbyn M. (2016). Triage of HPV positive women in cervical cancer screening. J. Clin. Virol..

[9] Bonde J., Floore A., Ejegod D., Vink F.J., Hesselink A., van de Ven P.M. (2021). Methylation markers FAM19A4 and miR124-2 as triage strategy for primary human papillomavirus screen positive women: a large European multicenter study. Int. J. Cancer.

[10] Kong L., Wang L., Wang Z. (2020). DNA methylation for cervical cancer screening: a training set in China. Clin. Epigenet..

[11] Adcock R., Nedjai B., Lorincz A.T., Scibior-Bentkowska D., Banwait R., Torrez-Martinez N. (2022 Oct 1). DNA methylation testing with S5 for triage of high-risk HPV positive women. Int. J. Cancer.

[12] Lorincz A.T., Brentnall A.R., Scibior-Bentkowska D., Reuter C., Banwait R., Cadman L. (2016). Validation of a DNA methylation HPV triage classiﬁer in a screening sample. Int. J. Cancer.

[13] Boers A., Wang R., van Leeuwen R.W., Klip H.G., de Bock G.H., Hollema H. (2016). Discovery of new methylation markers to improve screening for cervical intraepithelial neoplasia grade 2/3. Clin. Epigenet..

[14] Molano M., Tabrizi S.N., Garland S.M., Roberts J.M., Machalek D.A., Phillips S. (2016). CpG methylation analysis of HPV16 in Laser capture microdissected archival tissue and whole tissue sections from high grade anal squamous intraepithelial lesions: a potential disease biomarker. PLoS One.

[15] Li D., Xie Z., Pape M.L., Dye T. (2015 Jul 10). An evaluation of statistical methods for DNA methylation microarray data analysis. BMC Bioinf..

[16] Vallely A.J.B., Saville M., Badman S.G., Gabuzzi J., Bolnga J., Mola G.D.L. (2022 Sep). Point-of-care HPV DNA testing of self-collected specimens and same-day thermal ablation for the early detection and treatment of cervical pre-cancer in women in Papua New Guinea: a prospective, single-arm intervention trial (HPV-STAT). Lancet Global Health.

[17] Phillips S., Cassells K., Garland S.M., Machalek D.A., Roberts J.M., Templeton D.J. (2022 Mar 3). Gene methylation of CADM1 and MAL identified as a biomarker of high grade anal intraepithelial neoplasia. Sci. Rep..

[18] Vasiljevic N., Scibior-Bentkowska D., Brentnall A.R., Cuzick J., Lorincz A.T. (2014). Credentialing of DNA methylation assays for human genes as diagnostic biomarkers of cervical intraepithelial neoplasia in high-risk HPV positive women. Gynecol. Oncol..

[19] Molano M., Garland S.M., Cornall A.M. (2018). Laser microdissection for human papillomavirus (HPV) genotyping attribution and methylation pattern analyses of squamous intraepithelial lesions. Methods Mol. Biol..

[20] Thiele C., Hirschfeld G. (2021). Cutpointr: improved estimation and validation of optimal cutpoints in R. J. Stat. Software.

[21] Team R.C.R. (2018).

[22] Dafou D., Grun B., Sinclair J., Lawrenson K., Benjamin E.C., Hogdall Estrid (2010). Microcell-mediated chromosome transfer identifies EPB41L3 as a functional suppressor of epithelial ovarian cancers. Neoplasia Press, Inc..

[23] Schulz W.A., Alexa A., Jung V., Hader C., Hoffmann M.J., Yamanaka M. (2007). Factor interaction analysis for chromosome 8 and DNA methylation alterations highlights innate immune response suppression and cytoskeletal changes in prostate cancer. Mol. Cancer.

[24] Khongsti S., Shunyu B.N., Ghosh S. (2019). Promoter-associated DNA methylation & expression profiling of genes (FLT 3, EPB41L3 & SFN) in patients with oral squamous cell carcinoma in the Khasi & Jaintia population of Meghalaya, India. Indian J. Med. Res..

[25] Sha Y., Liu Y., Yang X., Wang J., Zhang R., Shen F. (2024). Exploring the diagnostic potential of *EPB41L3* methylation in cervical cancer and precancerous lesions: a systematic review and meta-analysis. Gynecol. Obstet. Invest..

[26] Eijsink J.J.H., Lendvai Á., Deregowski V., Klip H.G., Verpooten G., Dehaspe L. (2012). A four-gene methylation marker panel as triage test in high-risk human papillomavirus positive patients. Int. J. Cancer.

[27] Clarke M.A., Luhn P., Gage J.C., Bodelon C., Dunn S.T., Walker J. (2017). Discovery and validation of candidate host DNA methylation markers for detection of cervical precancer and cancer. Int. J. Cancer.

[28] van Leeuwen R.W., Ostrbenk A., Poljak M., van der Zee A.G.J., Schuuring E., Wisman G.B.A. (2019). DNA methylation markers as a triage test for identification of cervical lesions in a high risk human papillomavirus positive screening cohort. Int. J. Cancer.

[29] Gilham C., Nedjai B., Scibior-Bentkowska D., Reuter C., Banwait R., Brentnall A.R. (2024). Long-term prediction by DNA methylation of high-grade cervical intraepithelial neoplasia: results of the ARTISTIC cohort. Int. J. Cancer.

[30] Kelly H.A., Chikandiwa A., Warman R., Segondy M., Sawadogo B., Vasiljevic N. (2018). Associations of human gene EPB41L3 DNA methylation and cervical intraepithelial neoplasia in women living with HIV-1 in Africa. AIDS.

[31] Li N., Hu Y., Zhang X., Liu Y., He Y., van der Zee A.G.J. (2021). DNA methylation markers as triage test for the early identification of cervical lesions in a Chinese population. Int. J. Cancer.

[32] Brentnall A.R., Vasiljevic N., Scibior-Bentkowska D., Cadman L., Austin J., Cuzick J. (2015). HPV33 DNA methylation measurement improves cervical pre-cancer risk estimation of an HPV16, HPV18, HPV31 and EPB41L3 methylation classifier. Cancer Biomarkers.

[33] Wang H., Xu M., Cui X., Liu Y., Zhang Y., Sui Y. (2016). Aberrant expression of the candidate tumor suppressor gene DAL-1 due to hypermethylation in gastric cancer. Sci. Rep..

[34] Ma X., Liu J., Wang H., Jiang Y., Wan Y., Xia Y. (2020). Identification of crucial aberrantly methylated and differentially expressed genes related to cervical cancer using an integrated bioinformatics analysis. Biosci. Rep..

[35] Boers A., Bosgraaf R.P., van Leeuwen R.W., Schuuring E., Heideman D.A., Massuger L.F. (2014). DNA methylation analysis in self-sampled brush material as a triage test in hrHPV positive women. Br. J. Cancer.

[36] de Waard J., Bhattacharya A., de Boer M.T., van Hemel B.M., Esajas M.D., Vermeulen K.M. (2023). Identification of a methylation panel as an alternative triage to detect CIN3+ in hrHPV-positive self-samples from the population-based cervical cancer screening programme. Clin. Epigenet..

[37] de Wilde J., Kooter J.M., Overmeer R.M., Claassen-Kramer D., Meijer C.J., Snijders P.J. (2010). hTERT promoter activity and CpG methylation in HPV-induced carcinogenesis. BMC Cancer.

[38] Jiang J., Zhao L.J., Zhao C., Zhang G., Zhao Y., Li J.R. (2012). Hypomethylated CpG around the transcription start site enables TERT expression and HPV16 E6 regulates TERT methylation in cervical cancer cells. Gynecol. Oncol..

[39] Schutze D.M., Snijders P.J., Bosch L., Kramer D., Meijer C.J., Steenbergen R.D. (2013). Differential in vitro immortalization capacity of eleven (probably) high-risk human papillomavirus types. J. Virol..

[40] Schütze D.M., Kooter J.M., Wilting S.M., Meijer C.J., Quint W., Snijders P.J. (2015). Longitudinal assessment of DNA methylation changes during HPVE6E7-induced immortalization of primary keratinocytes. Epigenetics.

[41] Molano M., Moreno-Acosta P., Morales N., Burgos M., Buitrago L., Gamboa O. (2016). Association between type-specific HPV infections and hTERT DNA methylation in patients with invasive cervical cancer. Cancer Genomics Proteomics.

[42] Iliopoulos D., Oikonomou P., Messinis I., Tsezou A. (2009). Correlation of promoter hypermethylation in hTERT, DAPK and MGMT genes with cervical oncogenesis progression. Oncol. Rep..

[43] Eijsink J.J., Yang N., Lendvai A., Klip H.G., Volders H.H., Buikema H.J. (2011 Feb). Detection of cervical neoplasia by DNA methylation analysis in cervico-vaginal lavages, a feasibility study. Gynecol. Oncol..

[44] Li H., Liu J.P. (2007). Mechanisms of action of TGF-beta in cancer: evidence for Smad3 as a repressor of the hTERT gene. Ann. N. Y. Acad. Sci..

[45] Cassar L., Nicholls C., Pinto A.R., Chen R., Wang L., Li H. (2017). TGF-beta receptor mediated telomerase inhibition, telomere shortening and breast cancer cell senescence. Protein Cell.

[46] Choi J.H., Park S.H., Park J., Park B.G., Cha S.J., Kong K.H. (2007). Site-specific methylation of CpG nucleotides in the hTERT promoter region can control the expression of hTERT during malignant progression of colorectal carcinoma. Biochem. Biophys. Res. Commun..

[47] Dankai W., Khunamornpong S., Siriaunkgul S., Soongkhaw A., Janpanao A., Utaipat U. (2019). Role of genomic DNA methylation in detection of cytologic and histologic abnormalities in high risk HPV infected women. PLoS One.

[48] Steenbergen R.D., Ongenaert M., Snellenberg S., Trooskens G., van der Meide W.F., Pandey D., Bloushtain-Qimron N., Polyak K., Meijer C.J., Snijders P.J., Van Criekinge W. (2013 Sep). Methylation-specific digital karyotyping of HPV16E6E7-expressing human keratinocytes identifies novel methylation events in cervical carcinogenesis. J. Pathol..

[49] Luttmer R., De Strooper L.M.A., Dijkstra M.G. (2016). Comparing the performance of FAM19A4 methylation analysis, cytology and HPV16/18 genotyping for the detection of cervical (pre)cancer in high-risk HPV-positive women of a gynecologic outpatient population. Int. J. Cancer.

[50] Kremer W.W., Steenbergen R., Heideman D., Kenter G.G., Meijer C. (2021). The use of host cell DNA methylation analysis in the detection and management of women with advanced cervical intraepithelial neoplasia: a review. BJOG.

[51] Vink F.J., Lissenberg-Witte B.I., Meijer C.J.L.M., Berkhof J., van Kemenade F.J., Siebers A.G. (2021). FAM19A4/miR124-2 methylation analysis as a triage test for HPV-positive women: cross-sectional and longitudinal data from a Dutch screening cohort. Clin. Microbiol. Infect..

[52] Al Roomy M., Chehadeh W., Al Awadhi R. (2023). Prediction of cervical cancer precursor lesions by quantitative methylation specific PCR: a retrospective study. Cytopathology.

[53] De Strooper L.M.A., Verhoef V.M.J., Berkhof J., Hesselink A.T., de Bruin H.M.E., van Kemenade F.J. (2016). Validation of the FAM19A4/mir124-2 DNA methylation test for both lavage- and brush-based self-samples to detect cervical (pre)cancer in HPV-positive women. Gynecol. Oncol..

[54] de Waard J., Bhattacharya A., de Boer M.T., van Hemel B.M., Esajas M.D., Vermeulen K.M. (2024). Methylation analysis to detect CIN3+ in hrHPV-positive self-samples from the population-based cervical cancer screening program. Mod. Pathol..

